# Hedgehog Signaling in Malignant Pleural Mesothelioma

**DOI:** 10.3390/genes6030500

**Published:** 2015-07-08

**Authors:** Emanuela Felley-Bosco, Isabelle Opitz, Mayura Meerang

**Affiliations:** 1 University Hospital Zurich, Laboratory of Molecular Oncology, Clinic of Oncology, Haeldeliweg 4, 8044 Zürich, Switzerland; 2 University Hospital Zurich, Division of Thoracic Surgery, Raemistrasse 100, 8091 Zurich, Switzerland; E-Mails: isabelle.schmitt-opitz@usz.ch (I.O.); mayura.meerang@usz.ch (M.M.)

**Keywords:** Hedgehog signaling, malignant pleural mesothelioma, Gli-1, desert Hedgehog, HHip, TCGA, autocrine signaling, paracrine signaling

## Abstract

Malignant pleural mesothelioma (MPM) is a cancer associated with exposure to asbestos fibers, which accumulate in the pleural space, damage tissue and stimulate regeneration. Hedgehog signaling is a pathway important during embryonic mesothelium development and is inactivated in adult mesothelium. The pathway is reactivated in some MPM patients with poor clinical outcome, mainly mediated by the expression of the ligands. Nevertheless, mutations in components of the pathway have been observed in a few cases. Data from different MPM animal models and primary culture suggest that both autocrine and paracrine Hedgehog signaling are important to maintain tumor growth. Drugs inhibiting the pathway at the level of the smoothened receptor (Smo) or glioma-associated protein transcription factors (Gli) have been used mostly in experimental models. For clinical development, biomarkers are necessary for the selection of patients who can benefit from Hedgehog signaling inhibition.

## 1. Introduction: Malignant Pleural Mesothelioma

**Figure 1:**
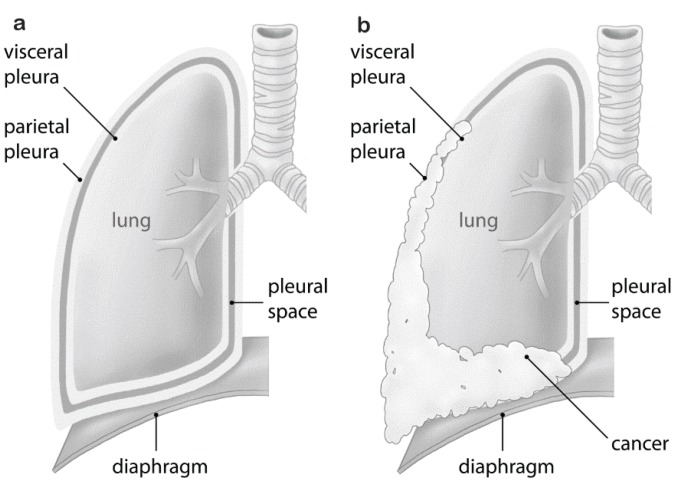
Malignant pleural mesothelioma *vs*. normal pleura: (**a**) normal pleura; (**b**) malignant pleural mesothelioma growth resulting in compression of the lung.

Malignant pleural mesothelioma (MPM) is an aggressive tumor arising from the mesothelial lining cells of the pleura (Figure 1). MPM is a rare cancer, difficult to treat and commonly associated with asbestos exposure (reviewed in [1]). Although Wagner had observed the association between asbestos and mesothelioma in 1960 [2], it took 30 years for the first regulatory measures to be implemented in developed countries, beginning in the United Kingdom (U.K.) and shortly thereafter the United States (U.S.). In many developing countries, asbestos is still being used. In Europe, the incidence of mesothelioma is about 20 per million with large intercountry variation [3]. Based on the Italian Mesothelioma Registry, median latency between asbestos exposure and disease onset is 44.6 years and increases over time in a linear fashion [4]. We therefore expect incidence rates in European nations still to be rising, with peak incidences around 2020 or later [5]. While about 80% of MPM cases are related to asbestos exposure [6], germline mutations of the BRCA1-associated protein-1 (*BAP1*) predispose individuals to MPM [7].

MPM is a very heterogeneous and highly chemoresistant tumor; therefore, management remains a clinical challenge. The best response rates are being reported after chemotherapy with cisplatin and pemetrexed [8]. Several aspects of mesothelioma treatment are under controversial discussion, in particular, extent and best type of surgery, need for radiotherapy and neoadjuvant as compared to adjuvant treatment. For less advanced disease, multimodality treatment, including neoadjuvant chemotherapy with cisplatin and pemetrexed, followed by extrapleural pneumonectomy with or without chemotherapy, is offered [9]. For patients with more advanced disease, the combination of cisplatin and pemetrexed has become the standard treatment, as supported by a phase 3 study [8].

Best survival data are reported from groups combining macroscopic complete resection achieved by extrapleural pneumonectomy or pleurectomy/decortication, chemo- and radio-therapy in a multimodal treatment [10,11,12,13]. However, the nature of the pleura makes it virtually impossible to resect with adequate safety margins. To increase the local tumor control and decrease recurrence, local adjuvant strategies have been developed. They include intracavitary treatment with photodynamic therapy [14] or chemotherapy [15,16].

For patients who cannot benefit from multimodality treatment, other treatment options, including immunotherapy, as well as targeting oncogenic pathways activated in MPM are under evaluation in the clinic (reviewed in [17]). One pathway that is activated in MPM and that we were the first to describe in this disease is the Hedgehog pathway (Hh) [18].

## 2. The Hedgehog Signaling Pathway in MPM

**Figure 2:**
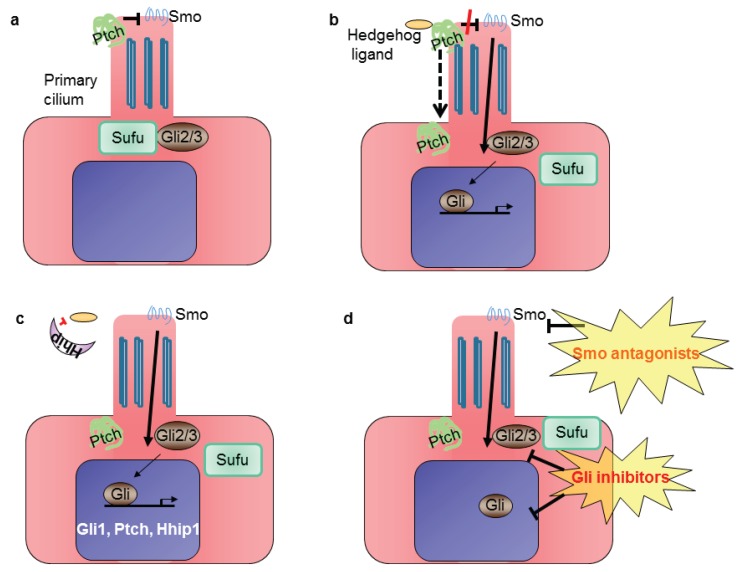
Simplified scheme of Hh signaling in mammalian cells: (**a**) in the absence of Hedgehog ligand, patched 1 (Ptch) localizes to the primary cilium, where it prevents activation of smoothened (Smo); (**b**) Hh signaling is activated upon binding of the ligand to the Ptch receptor, which leaves the cilium, releases the inhibition of Smo and leads to glioma-associated (Gli) transcription factors’ translocation into the nucleus; (**c**) Gli activates the expression of target genes *Gli1*, *Ptch* and *Hedgehog interacting protein* (*Hhip1*); (**d**) the pathway can be inhibited at the level of Smo or Gli.

Classical Hh core signaling components (reviewed in [19]) are the Hedgehog ligands (Sonic Hedgehog, Shh; desert Hedgehog, Dhh; Indian Hedgehog, Ihh), which upon binding to the transmembrane receptor patched (Ptch) causes Ptch to remove its inhibitory influence on the G protein-coupled receptor smoothened (Smo) (Figure 2). Activation of Smo then leads to nuclear translocation of the glioma-associated protein (Gli) family of transcription factors and induction of Hh target genes, such as *Gli1* and Hedgehog interacting protein (*Hhip*). The latter competes with Ptch by binding to Hh ligands [20]. Gli protein levels and activities are primarily regulated by suppressor of fused (Sufu). Sufu is a major negative regulator of mammalian Hh signaling. Loss of Sufu in mammals leads to global Hh pathway activation and early embryonic lethality [21,22]. In the absence of the Hedgehog family of ligands, Gli-mediated transcription is inhibited. In vertebrates, Hh signaling occurs in the primary cilium, a non-motile flagellar-like organelle present on growth-arrested cells [23].

The Hh signaling pathway is essential for embryonic mesothelial development [24]. In the adult, Hh continues to signal to discrete populations of stem and progenitor cells within various organs where it is considered to have a role in maintaining homeostasis after injury (reviewed in [19]). The Hh signaling pathway is inactive in most adult tissues, including mesothelium [18], but consistent with re-activation of developmental pathways in cancer, the Hh pathway has been shown to become upregulated in MPM tumors. We were the first to show *SHH* gene expression in human MPM tumor tissues along with increased expression levels of *HHIP* and *GLI1* [18]. High *GLI1* gene expression was associated with shorter overall survival in MPM patients [18]. Another study reported that high SMO and SHH expression levels were associated with worse survival of MPM patients [25].

The importance of the Hh pathway in MPM has been recently reinforced by data obtained in samples from 85 patients profiled for molecular differences between tumor cells and healthy cells by large consortia, such as The Cancer Genome Atlas (TCGA). Since 2006, TCGA has explored these differences in many cancer types using a variety of platforms using single-nucleotide polymorphism, small RNA transcriptome, exome and methylation data from sequencing and microarrays. Systematic analysis of TCGA datasets was generated through the Broad’s Genome Data Analysis Center (GDAC; http://gdac.broadinstitute.org/). The pipelines run in a computational framework called Firehose, which also generates analysis reports [26]. The Hh pathway appears in the top 10 pathways deregulated in MPM (Table 1) [27]. Moreover, based on the mRNA expression profile, the 85 tumors clustered into four groups [28], one of which is characterized by overexpression of Hh target genes *GLI1*, *HHIP* and *PTCH2*. For 39 of these patients, clinical outcome is also available, and the Hh target gene-expressing group is one of the two groups with the worst overall survival (median 6.6 months, [29]).

**Table 1:** Malignant pleural mesothelioma (MPM) specific pathway perturbations reported in The Cancer Genome Atlas (TCGA).

Pathway Name	Average No. of Perturbations
WT1 (Nephrin/Neph1/Yap/TEAD)	18
Ephrin A reverse signaling	13
Syndecan-1-mediated signaling	11
Hedgehog signaling	10
IL-4-mediated signaling	10
Signaling regulated by Ret	10
Ephrin B reverse signaling	10
Endothelins	10
CDKN2A (Rb pathway)	10
Glucocorticoid receptor regulatory network	9

Altogether, published work and data mining indicate that deregulated Hh signaling defines a subgroup of patients with bad clinical outcome.

With the exception of the fact that Hedgehog signaling is essential for embryonic mesothelium development and that re-activation of embryonic signaling is frequently observed in tumors, the factors driving aberrant Hh pathway activity in MPM are not known yet. A study using a panel of seven MPM cell lines demonstrated a missense mutation in the *SUFU* gene, leading to T411M amino acid change, coupled with three base pair (CTG) insertions in the *SMO* gene, resulting in an additional amino acid 23L_24GinsL in the signal peptide region in one cell line [30]. The 3-bp insertion in *SMO* was also detected in one MPM patient out of 14 patients analyzed. Transfection of a *SUFU* cDNA harboring the T411M missense mutation suppressed Gli-reporter gene downregulation as observed with wild-type *SUFU* [30], although it is unclear whether such results were observed with similar levels of SUFU protein expressed. Deletion of *PTCH1* exons 8–23 was observed in another cell line [30]. Deletion of chromosome 9q22.32, containing the *PTCH1* gene, is observed in medulloblastoma and basal cell carcinoma (reviewed in [31]) and various other cancers [32,33,34], consistent with the loss of tumor suppressor function. The functional impact of the insertion in the *SMO* gene still remains to be investigated. This data indicate that mutations of the Hh pathway rarely exist in MPM.

As mentioned above, the Hh pathway is important for development and tissue repair, and it is also active in cancer cells with the stem cell phenotype (reviewed in [35,36]). We had hypothesized that cancer cells with the stem cell phenotype are present in MPM, a cancer type with a high chemoresistant and relapse rate. Therefore, we used a functional assay, which identifies a small and distinct subset of cells, called a “side population” (SP), with phenotypic markers of multipotent hematopoietic stem cells after staining bone marrow with the DNA staining dye Hoechst 33342 [37]. The SP is due to the expression of functional ATP-binding cassette (ABC) transporters [38]. When living cells are stained with Hoechst 33342, SP cells efflux the DNA staining dye via their ABC transporters. When cells are co-incubated with ABC transporter inhibitors verapamil or fumitremorgin C (FTC), Hoechst 33342 is no longer effluxed, leading to a shift in the dual emission wavelength fluorescence-activated cell sorting (FACS) analysis upon which the SP can be identified. The ABCG2 drug transporter is responsible for the SP in the bone marrow [38,39]. We detected side population cells of MPM having precursor characteristics and showing the enrichment of tumor initiating and *PTCH1* expression compared to the non-side population [40].

**Figure 3:**
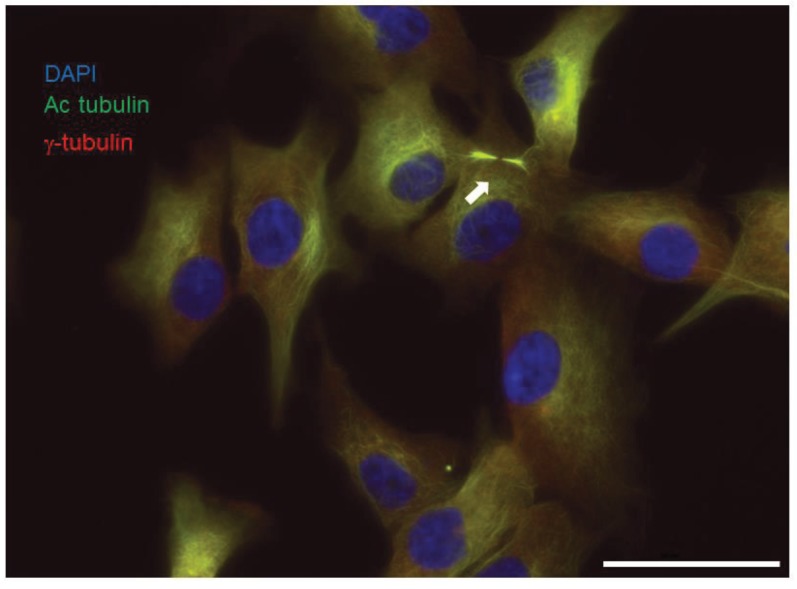
Primary culture of mesothelioma grown in the absence of serum and in 3% oxygen develop primary cilia. Primary mesothelioma cells were fixed in 4% paraformaldehyde, then were permeabilized in 0.1% saponin. Primary cilia (arrow) were detected using anti-acetylated tubulin (Sigma T 6793, 1:50), anti-gamma tubulin (Sigma T 5192, 1:1000) and Alexa 488 and Cy5-coupled secondary antibodies, respectively. Nuclei were stained with DAPI. Scale bar: 50 μm.

Stem cell signaling is, as also observed by others [41], not maintained when primary tumors are grown in the presence of serum; therefore, we set up primary human MPM in the absence of serum and in the presence of their own conditioned medium plus specific growth factors [42,43] and in 3% oxygen. The culture conditions in 3% instead of 20% oxygen were implemented because they are more physiologic [44]. In these conditions, we could observe the presence of primary cilia (Figure 3), probably linked to the fact that about 35% of the cells are quiescent [45]. Autocrine regulation of the Hh pathway in a ligand-dependent fashion was also observed [18]. Indeed, secretion of desert Hedgehog ligand (DHH) was observed to stimulate MPM growth by regulating Yes-associated protein 1, a transcription co-activator controlled by the Hippo pathway [18]. Consistent with the Hh pathway being overexpressed in a subgroup of MPM as detailed above, only four out of six of the primary MPM cell lines cultured in these conditions responded to the inhibition of the Hh pathway by the Smo antagonist, cyclopamine. The treatment with another Smo antagonist, HhAntag, increased the number of growth-arrested cells in an Hh-responsive cell line *in vitro* [45] and reduced the tumor growth implanted subcutaneously in immunodeficient mice by approximately 40% [18]. Thus, the Hh pathway activation in MPM may originate from increased pathway activity in a ligand-dependent manner during the regeneration and repair of mesothelium after tissue damage. Nevertheless, it should be noted that a small group of MPM patients may harbor mutations of the pathway.

In addition, we recently detected paracrine activation of Hh signaling in MPM. Analysis of GLI1 immunoreactivity in a small cohort of MPM patients revealed heterogeneous expression in both tumor and stroma fractions [46]. In order to investigate in the most complete way the role of Hh signaling in both tumor and stroma, we used a rat immunocompetent mesothelioma model showing activation of Hh signaling [46]. We observed downregulation of the Hh pathway mostly in the stromal part after the treatment with an Hh antagonist [46]. Downregulation of the Hh pathway was associated with reduced expression of factors essential for promoting tumor growth and with reduced tumor volume [46]. The same finding has been detected in pancreatic and colorectal cancers [47,48,49]. The role of the stromal Hh pathway in tumor maintenance was clearly demonstrated in pancreatic cancer, where tumor growth was reduced when growing in a microenvironment lacking Gli1 compared to the wild-type [49]. The role of the microenvironment should not be neglected, especially in MPM, given the number of MPM-specific pathway perturbations associated with the tumor to stroma interaction reported in The Cancer Genome Atlas (TCGA) (Table 1).

A number of clinical trials for Hh inhibitors, mainly Smo antagonists, have been conducted to date (reviewed in [36,50]). Three mesothelioma patients were enrolled in the phase 1 clinical trial for Smo inhibitor vismodegib and showed no response [51]; however, in that study, activation of the Hh pathway in the tumors was not investigated. As for other targeted therapy, there is a need for proper predictive biomarkers. In medulloblastoma, another cancer where a subgroup of patients show Hh activation, a five-gene expression signature was used to select patients who received Hh inhibitor, and 66% showed objective responses [52]. A phase 3 trial in patients with recurrent medulloblastoma is ongoing to validate this assay for predicting the response to Hh pathway inhibition. It is not known whether this gene signature would be the same in mesothelioma.

Several lines of evidence suggest existing crosstalk between Gli1 and other signal transduction pathways (reviewed in [53]) in an Hh ligand-independent manner. For example, an activated mTOR pathway, which we and others have shown to be associated with the worst clinical outcome in mesothelioma [54,55], promotes Gli1 transcriptional activity [56]. It is not clear yet what the role of non-canonical Hh activation in MPM is. Nevertheless, novel therapies targeting the Hedgehog pathway downstream of Smo have been developed. Several studies reported that Gli1 inhibition, either by agents, such as arsenic trioxide, which prevents Gli2 localization to primary cilia [57], inhibits GLI1 transcriptional activity [58] and suppresses Gli transcription via induction of DNA damage [59] or GANT61, which prevents Gli1-DNA binding in living cells [60], but it also has many other mechanisms of action (reviewed in [61]), resulting in growth arrest and induction of cell death in MPM cells *in vitro* [62,63,64].

## 3. Conclusions

In summary, several lines of evidence indicate that Hh signaling is activated in a subgroup of MPM patients and is associated with the worst clinical outcome. In a limited number of them, activation may result from either mutation in components of the pathway, while for the others, it seems to be resulting from ligand-driven activation. The identification of biomarkers and parallel or compensatory non-canonical signaling may be useful for exploring mechanism-based therapeutic combinations.
